# Further Insights into the Measurement of Free Polysaccharide in Meningococcal Conjugate Vaccines

**DOI:** 10.3390/vaccines13020167

**Published:** 2025-02-09

**Authors:** Nicola J. Beresford, Gianluigi De Benedetto, Kay Lockyer, Fang Gao, Karena Burkin, Karan Lalwani, Barbara Bolgiano

**Affiliations:** Science, Research and Innovation Division, Medicines and Healthcare Products Regulatory Agency, South Mimms EN6 3QG, UK; gianluigi.debenedetto@reithera.com (G.D.B.); kay.lockyer@mhra.gov.uk (K.L.); fang.gao@biojw.com (F.G.); k.lalwani01@gmail.com (K.L.)

**Keywords:** CRM, filtration, glycoconjugate, HPAEC, HPLC, meningitis, meningococcal, *Neisseria meningitidis*, stability

## Abstract

**Objectives**: The purpose of this study was to further characterize the ultrafiltration (UF) method for determining free saccharide levels in glycoconjugate vaccines and compare it with other methods used for the determination of free saccharide levels in meningococcal glycoconjugate vaccines. **Methods**: We performed experiments on both meningococcal glycoconjugates and capsular polysaccharides, and compared UF, deoxycholate (DOC) precipitation, and solid-phase extraction (SPE) methods. Meningococcal capsular polysaccharides from groups A (MenA), C (MenC), and W (MenW) were depolymerized and characterized using SEC-MALS (size-exclusion chromatography with multi-angle laser light scattering) to determine the molecular weight and hydrodynamic size and then subjected to UF. The free saccharide content was quantified using HPAEC-PAD (high-performance anion-exchange chromatography with pulsed amperometric detection). **Results**: The characterization of size-reduced group C polysaccharide revealed weight-average molecular mass (Mw) ranging from 22,200 g/mol to 287,300 g/mol and hydrodynamic radii of 3.7 to 19.5 nm. Pore size studies confirmed that polysaccharides with diameters up to 15 nm filtered through the 100 kDa cellulose membrane. The smallest PS fragment tested (22,200 g/mol, 7.4 nm diameter) was partially recovered from the 30 kDa membrane. For MenC-CRM_197_, DOC yielded the lowest free saccharide content (<1%), UF gave moderate results (7–8%), and SPE showed the highest and most variable values (up to 15%). For MenA- and MenW-CRM_197_, UF and DOC consistently provided low free saccharide levels (<2% and 3–11%, respectively). **Conclusions:** The upper limits on the size of free group C meningococcal polysaccharides that can be ultrafiltered were assessed. Differences in the relative amount of free saccharide were observed between various methods used to control meningococcal conjugate vaccines.

## 1. Introduction

The effectiveness of glycoconjugate vaccines in protecting against diseases caused by encapsulated bacteria depends on generating bactericidal antibodies specific to the polysaccharide (PS) constituting the bacterial capsule [1,2,3]. This requires preserving essential immunogenic saccharide–protein epitopes, maintaining an optimal PS or oligosaccharide (OS) chain length, minimizing structural modifications, and securing efficient conjugation of PS or OS to a well-tolerated T-cell-dependent carrier protein [4,5]. Certifying the stability and integrity of the conjugate in both the drug substance and the final product is one of the most critical elements [6]. The vaccine’s potency is directly related to the effective amount of saccharide that is covalently attached to the carrier protein. Free saccharide, which is not bound to the protein, may remain as a residual from purification or may form due to PS instability as a result of hydrolysis or depolymerization. Only the conjugated saccharide is essential for clinical protection, and high levels of free saccharide result in a lower dose of conjugated saccharide and can also suppress the immune response. Therefore, each batch must be tested to ensure free saccharide levels remain within the specification limits during the manufacturing of upstream components to safeguard the vaccine’s efficacy throughout its shelf life [7].

The analytical evaluation of vaccines is critical to determine consistent quality and immunogenicity. For glycoconjugates, this involves testing starting materials, intermediates, and the final conjugate [8]. It is important to measure free PS levels in both the bulk conjugate and the final product during release and throughout stability studies. Maintaining low free saccharide levels across the product’s shelf life is critical for preserving vaccine potency. Stability-indicating tests are used to establish the relationship between vaccine quality, stability, and efficacy, and determine the product’s expiration date.

The separation of free saccharide from conjugated saccharide is the first step in its determination, using techniques based on size, charge, protein hydrophobicity, or antigenic specificity. These include chromatographic techniques such as size-exclusion, ion-exchange [9], reverse-phase [10], and hydrophobic interaction. Other methods involve chemical precipitation of the conjugate with acid and/or detergents [11,12,13], ammonium sulfate or aluminum adsorption; capillary electrophoresis [14,15], centrifugal ultrafiltration (UF) [16,17,18], ultracentrifugation [19], solid-phase extraction (SPE) [20,21]; and antibody capture and immunoprecipitation [22]. Once separated, free saccharide is then quantified using chemical and immunological methods similar to those employed for measuring total saccharide content, including high-performance liquid chromatography (HPLC), high-performance anion-exchange chromatography with pulsed amperometric detection (HPAEC-PAD) [16,23,24,25], gas chromatography (GC) after hydrolysis [26], colorimetric methods [27], and ELISA [22,28].

The ratio of free to total saccharide is a critical parameter for evaluating the effectiveness of the purification process and the integrity of the glycoconjugate. Validation of the chosen method is essential, especially given challenges such as electrostatic interactions and the diverse range of polysaccharides with respect to the carrier protein or conjugate. Forced degradation studies conducted during vaccine development can further confirm the method’s suitability. Difficulties may occur when applying these methods to final formulations that contain stabilizing sugars or adjuvants, which can complicate the accurate measurement of specific saccharides. Combination vaccines with similar monosaccharide components present additional difficulties [29,30].

The ultrafiltration of proteins for the purpose of purification and characterization was developed on a commercial scale in the 1970s with Amicon Dia-Flo membranes, which were semipermeable membranes with different chemical characteristics that were used with external pressure or centrifugal force. Applications were later extended to polysaccharides and nucleic acids. Such size-based separations can also be applied to measure the unconjugated or “free” saccharide arising from small oligosaccharides or monosaccharides in a glycoconjugate vaccine [16,17].

In our laboratory, the level of free saccharide separated using a UF approach was shown to correlate with the serum bactericidal activity in meningococcal group A conjugate vaccines [31] and with vaccine immunogenicity in meningococcal groups A, C, W, X, and Y subjected to thermal stability studies [17,31,32]. The objective of this study was to further characterize the ultrafiltration membrane method using sized PS fragments and to compare it with commonly used methods, such as detergent precipitation and hydrophobic bead binding, which rely on the hydrophobicity of the conjugated carrier protein. The effect of ionic strength on the free saccharide determination of MenC-CRM_197_ (meningococcal group C–cross-reacting material 197) [33] was also explored.

## 2. Materials and Methods

### 2.1. Polysaccharides

The source of the meningococcal group C PS was the 1st WHO International Standard (IS), NIBSC 08/214 [34]. Sized polysaccharides were prepared for the characterization of ultrafiltration membranes using mild acid hydrolysis, followed by purification via HPLC size-exclusion chromatography (HPLC-SEC).

The MenC PS (1 mg/mL) was depolymerized by incubation in 50 mM sodium acetate buffer (pH 4.7 ± 0.05) at 73 °C ± 2 °C for 15, 30, and 90 min, as described by Bardotti et al. [35]. Cooled samples were neutralized with an equivalent volume of 25 mM sodium hydroxide, vortexed, and stored at 4 °C before concentration and desalting using Pall Microsep Advance 3 kDa MWCO Omega centrifugal devices for 15 min at 4400× *g*. Retentates were washed three times with deionized water and freeze-dried overnight using a ModulyoD freeze dryer (Thermo Electron, Crawley, UK).

Lyophilized samples were dissolved in 0.22 μm filtered PBS “A” (10.1 mM Na_2_HPO_4_, 1.84 mM KH_2_PO_4_, 171 mM NaCl, 3 mM KCl, pH 7.3–7.5) and injected (approximately 800 μg) onto a Tosoh TSK PW_XL_ guard column connected in series to a Tosoh TSKgel G4000 PW_XL_ HPLC column using a Thermo Scientific Ultimate 3000 HPLC system (Dionex, Sunnyvale, CA, USA). The system was equipped with a multi-wavelength UV-Vis detector set at 214, 260, and 280 nm and a differential refractive index (dRI) detector (Knauer, Berlin, Germany) set at 35 °C. The autosampler was maintained at 4 °C, and the column compartment was set at 30 °C.

Samples were eluted at 0.250 mL/min with 0.22 μm filtered PBS “A” (60 min runtime). The void (V_0_) and total (V_t_) column volumes were determined using salmon DNA (Sigma Aldrich, St. Louis, MO, USA) and tyrosine (Sigma Aldrich), detected at 280 nm. Fractions were collected using the U3000 autosampler/fraction collector, collecting at 125 μL per fraction (30 s per fraction) into polypropylene autosampler vials and stored at −20 °C before being pooled. Untreated (full-length, FL) MenC PS was also desalted as described above.

A purified PS with relatively smaller size dimensions (Mw 24,100 g/mol; RMS Rn 8.4 nm; Rh(v) 13.1 nm), designated as PS4 in Lockyer et al. [36], was used as a control in free saccharide assays.

### 2.2. Glycoconjugate Vaccines

Six meningococcal-CRM_197_ glycoconjugate preparations were sourced from a vaccine manufacturer and used to evaluate two different free saccharide separation methods. These included two lots of meningococcal group A OS (MenA) CRM_197_ conjugate (designated A and B), two lots of meningococcal group C OS (MenC) CRM_197_ conjugate (designated C and D), and two lots of meningococcal group W OS (MenW) CRM_197_ conjugate (designated E and F).

### 2.3. Saccharide Analysis

The MenC content of the PS and the MenA, MenC, or MenW content of the glycoconjugate samples were determined by HPAEC-PAD [25,31] using the 1st WHO International Standards for MenA (NIBSC 13/246), MenC (NIBSC 08/214) [34], and MenW (NIBSC 16/152) polysaccharides (https://nibsc.org/, accessed on 29 January 2025) [25].

### 2.4. Characterization of the MenC Polysaccharide Fragments

The *O*-acetylation content of MenC PS was measured using NMR [37] and the micro-Hestrin colorimetric assay [38]. Sizing analysis by size-exclusion chromatography with multi-angle laser light scattering (SEC-MALS, Wyatt, Santa Barbara, CA, USA) was performed according to Lockyer et al. [38], using analytical HPLC columns in series: TSK guard, G6000 PW_XL_, and G5000 PW_XL_. The weight-average molecular mass (Mw, g/mol), polydispersity (Mw/Mn), and RMS radius moments (Rn, nm) values were determined, with a dn/dc value of 0.171 mL/g.

### 2.5. Ultrafiltration for Free Saccharide Determination

To determine the % recovery of the sized polysaccharides through ultrafiltration membranes for the purpose of exploring the permeability of the membranes, samples were diluted to 20 μg saccharide/mL in dH_2_O or PBS ‘A’, pH 7.3–7.5 buffer, and ultrafiltered using a pre-washed (three times with 0.5 mL dH_2_O, 10 min spin) Nanosep 3K Omega filter (PALL Life Science, Port Washington, NY, USA) or Microcon-10, -30, or -100 Ultracel YM-type regenerated cellulose filters (Millipore, Burlington, MA, USA) [17,31]. Ultrafiltration units containing samples were centrifuged at 7000× *g* for 15–20 min at room temperature, and filtrates were analyzed using HPAEC-PAD (Thermo Scientific Dionex, Sunnyvale, CA, USA) [25,31]. The sample for measuring the total saccharide (20 μg/mL) was not further filtered. Recovered and total polysaccharide samples were injected three and two times, respectively, and polysaccharide standards were run in triplicate, with two injections each. Percent recovery was calculated as the amount of saccharide in the filtrate divided by the amount of total saccharide in the sample. The % CV of MenC polysaccharide measured in different assays, based on percent recovery of the full-length IS from the filtrate, was 19% (30 kDa membrane) and 10% (with 100 kDa membrane) from four determinations with the 30 and 100 kDa membranes. The % CV of the total MenC saccharide in the IS was 4% from four different assays.

The UF method was also used for the purpose of testing whether an increase in ionic strength in the polysaccharide’s solute would increase the permeation of unconjugated saccharide from a sample of glycoconjugate, measured in the free saccharide assay performed to measure vaccine integrity. One lot of a MenC-CRM_197_ conjugate vaccine was filtered following dilution in water, or in 10 mM Bis-Tris, pH 7, containing 0, 5, 50, or 250 mM KCl, the buffer system used in the Zydney laboratory to study the effect of ionic strength [39,40,41]. In this case, free saccharide was separated from CRM_197_ conjugate vaccines using Microcon-30 filters, pre-washed as above, and centrifuged at 7000× *g* for 20 min. Percent free saccharide was calculated as the amount of saccharide in the filtrate following filtration of the acid hydrolysate (0.1 M HCl at 80 °C for 2 h) [31] divided by the total saccharide in the sample. A % CV for free saccharide was calculated from three sample preparations (replicates) of filtered saccharide, divided by the average of two replicates of total saccharide, with 1–9% intra-assay repeatability.

The final application of the UF method was performed for the purpose of comparing it with other methods for measuring free saccharide in a conjugate vaccine. Three serogroups of CRM_197_ conjugates, MenA, MenC, and MenW, were evaluated as manufactured bulk conjugates (two bulks for each serogroup). Filtration was performed with the 30 kDa membrane as per [17] and HPAEC-PAD according to [25,31]. Three replicates were injected twice, and two separate sessions were run. The % CV for repeatability was 0.1 (MenA), 0.65–1.4 (MenC), and 0.1 (MenW).

### 2.6. Deoxycholate (DOC) Precipitation for Free Saccharide Determination

To precipitate the meningococcal conjugate, the method was adapted from Lei et al. [12]. A total of 50 μL of freshly prepared 3% (*w*/*v*) sodium deoxycholate (pH ~8.2) was mixed with 450 μL of the sample at a concentration of 20 μg saccharide/mL. The samples were vortexed and incubated on ice for 30 min. Next, 25 μL of 1 N HCl was added and the mixture was vortexed again and then centrifuged at 10,000× *g* for 30 min. The supernatant was removed and analyzed using HPAEC-PAD. In the same format as the UF method, % CV for repeatability was 0.1–0.3 (MenA), 0.4 (MenC), and 0.6–3.2 (MenW).

### 2.7. Solid-Phase Extraction (SPE) for Free Saccharide Determination

To separate the free saccharide component of the meningococcal conjugate, samples were diluted to 5 μg saccharide/mL in dH_2_O. C4 columns (Grace-Vydac) were conditioned with methanol, washed with H_2_O, and equilibrated with 0.9% NaCl (pH 5.5) before sample addition. Free saccharides were eluted using NaCl solution and analyzed using HPAEC-PAD. The % CV for repeatability was 3.6–9.0 (MenC).

## 3. Results

### 3.1. Ultrafiltration Membrane Permeability to Group C Meningococcal Polysaccharides

In our laboratory, Amicon YM-type regenerated cellulose ultrafiltration membranes are used to separate unconjugated saccharide from conjugated saccharide to determine the percentage of free saccharide in glycoconjugate vaccines [17,31]. These membranes are low-binding, anisotropic, hydrophilic, and made of regenerated cellulose, available in various pore sizes defined by nominal molecular weight cutoff values (NMWCO). These cutoff values, expressed in approximate kDa, indicate the membrane’s ability to limit the permeability of proteins with the specified molecular weight. While this value is of use in selecting a membrane that will retain the conjugated carrier protein, such as CRM_197_ or tetanus toxoid (TT), it is less helpful in understanding the size at which PS will permeate or be retained.

**Figure 1:**
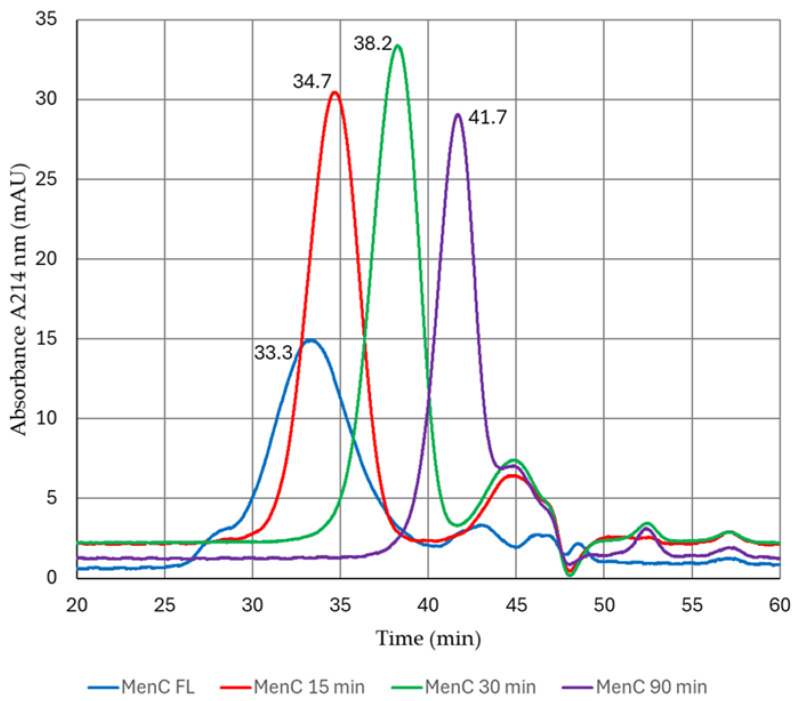
**SEC chromatograms of the purified MenC fractions after depolymerization and desalting.** MenC full-length PS (blue) was subjected to hydrolysis for 15 (red), 30 (green), and 90 min (purple). The injections were performed using a Tosoh TSK PW_XL_ guard column in series with Tosoh TSKgel G6000 + 5000 PW_XL_ HPLC columns with PBS “A” as the mobile phase. The void volume (V_0_) and total column volume (V_t_) were determined using salmon DNA (Sigma Aldrich) and tyrosine (Sigma Aldrich), measured at 24.6 and 49.9 min, respectively. The absorbance of the polysaccharides was measured at 214 nm (indicated on the y-axis), which detects their amine, carbonyl, and carboxyl groups. Peak elution times are displayed for each.

To further characterize the membranes for separating unconjugated PS, a panel of purified, sized MenC PS was prepared through mild acid hydrolysis to depolymerize full-length PS to varying extents. The size-exclusion chromatography (SEC) profiles following hydrolysis are shown in Figure 1.

The chromatogram of the full-length (FL) MenC PS was consistent with that previously reported by Vipond et al. [34], showing a single main peak with a leading shoulder and later eluting peaks beginning at approximately 42 min. The three PS fragments from this FL PS were produced following acid hydrolysis treatment for 15, 30, and 90 min. All peaks eluted as a single main peak, with successively later eluting peaks producing insignificant RI and light scattering (90°) signals.

The *O*-acetylation of these fragments was equivalent to the full-length PS [34], with values of 69% (FL), 57% (15 min), 66% (30 min), and 68% (90 min) obtained by the micro-Hestrin method, which can underestimate the content. NMR-determined *O*-acetylation confirmed a value of 95% for the FL [34].

The size and purity of the full-length MenC PS and its fragments were determined using SEC-MALS, as shown in Table 1. The full-length PS had a Mw of 287,300 g/mol and a hydrodynamic radius of 19.5 nm, and there was a successive decrease in the Mw and radius with each successive fragment. Like the full-length PS, the 15 and 30 min samples were monodisperse, with a Mw/Mn of 1.0–1.1. Only the smallest fragment showed higher polydispersity (1.5), perhaps reflecting the heterogeneity arising from the generation of depolymerized oligosaccharides under those conditions. Together, the panel comprised PS ranging from 22,2000 to 287,300 g/mol, with hydrodynamic radii of 3.7 to 19.5 nm.

**Figure 2:**
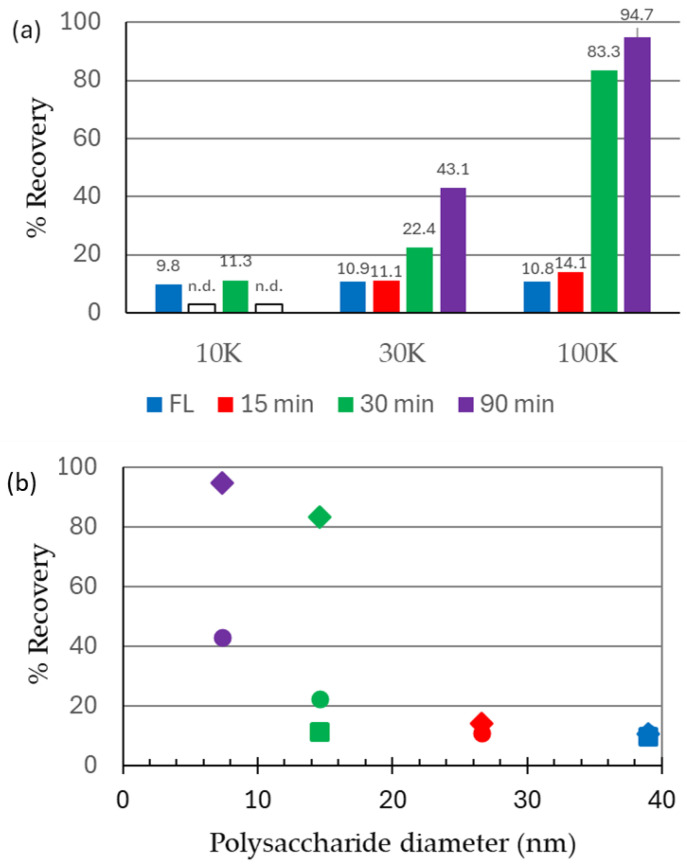
**Size characterization of ultrafiltration membranes using meningococcal group C polysaccharides**. (**a**) Recovery of sized MenC polysaccharides from Microcon-10, -30 kDa, and -100 kDa ultrafiltration membranes. Percent filtrate recovery values are listed on each bar. For two samples run with the 10 kDa membrane, n.d., no data were recovered. (**b**) Effect of PS diameter on PS recovery. Data points are from purified PS samples: full-length (blue), 15 min (red), 30 min (green), and 90 min (purple) hydrolysates. The recovery of PS from ultrafiltration membranes with the measured diameters from this study and NMWCO values: 10 kDa (squares), 30 kDa (circles), and 100 kDa (diamonds) membranes are shown.

This panel of polysaccharides was then tested for their recovery (or permeability) through ultrafiltration membranes with NMWCO values of 10 kDa, 30 kDa, and 100 kDa. Figure 2a illustrates the recovery of the polysaccharides from the Microcon-10, -30, and -100 membranes. The full-length (287,300 g/mol) and 15 min (130,000 g/mol) MenC PS samples were largely retained by the 30 kDa and 100 kDa membranes. Neither the full-length nor the 30 min (52,800 g/mol) PS samples permeated through the 10 kDa membrane (Figure 2a). The recovery of polysaccharides in the filtrates increased with larger pore sizes and smaller hydrodynamic volumes of the polysaccharides (Figure 2a,b). The 3 kDa PES membrane retained the FL PS (Figure 2b), with less than 1% recovery.

A separate study using atomic force microscopy determined the average pore sizes of these membrane types to be 8.7 nm for 3 kDa membranes, 11.3 nm for 10 kDa, 13.2 nm for 30 kDa, and 19.4 nm for 100 kDa membranes [42], with the range of sizes of pores extending to about ±50% of the mean.

Based on these pore sizes alone, it would be expected that the full-length PS (of 38.8 nm diameter) should be retained by all the membranes tested (0% recovery), while the smallest fragment (7.4 nm diameter) would permeate the two larger pore size membranes of 30 and 100 kDa NMWCO. This expectation was confirmed experimentally. Only polysaccharides with a diameter under 15 nm were able to pass through the 100 kDa membrane (mean pore size of 19 nm). The smallest PS (22,200 g/mol, 7.4 nm diameter) was almost fully recovered from the 100 kDa membrane, and 45% recovered from the 30 kDa membrane (Figure 2b).

### 3.2. Effect of Ionic Strength on Free Saccharide Content Determined by Ultrafiltration

The free saccharide content of a MenC-CRM_197_ conjugate lot, a measure of conjugate integrity, was determined after ultrafiltration on Microcon-30 centrifugal filters following dilution with varying ionic strength diluents. When the MenC-CRM_197_ conjugate was diluted to 20 µg/mL total saccharide in H_2_O, the free saccharide content was 7.5%. Then, the conjugate was diluted to the same saccharide concentration using 10 mM Bis-Tris buffer, pH 7, with increasing ionic strength (KCl). As shown in Table 2, no appreciable differences in free saccharide quantification with increasing ionic strengths were observed under the experimental conditions, with results ranging from 6.2 to 6.8% free saccharide.

### 3.3. Comparison of % Free Saccharide Determined by Ultrafiltration Compared with Detergent Precipitation and Hydrophobic Bead Methods

Three different methods for free saccharide analysis in glycoconjugate vaccines are widely used by vaccine manufacturers and quality control laboratories: UF, DOC, and SPE. Here, we evaluated these methods against the same MenC-CRM_197_ conjugate lots. Detergent precipitation by DOC yielded the lowest reported free saccharide levels at <1% for both conjugated samples tested (Table 3). UF with 30 kDa filters showed a modest free saccharide content of 7–8%, while the SPE method using C4 columns yielded significantly higher values of 15%. Good consistency was observed across replicates for both the UF and DOC methods, whereas the SPE method exhibited greater variability.

The low molecular weight (MW) MenC PS control (MenC 90 min) was a key component for distinguishing the differences between the methods. The almost full recovery (95%) of the PS in the supernatant of the control in the DOC method indicated that no PS is precipitated in the presence of the detergent. With the SPE method, approximately one-quarter of the material is lost during the extraction, indicating the interaction of the PS with the solid phase. The UF method applied to glycoconjugate vaccines showed a recovery of 20%, with the remaining being retained in the retentate of the filter. This indicates how the size of the PS fragment and the degradation pattern of glycoconjugate impact the overall result, with fragments of over 14 nm being retained by the filter.

The comparison between UF and DOC was further extended to other glycoconjugate groups, MenA-CRM_197_ and MenW-CRM_197_. The UF method gave the lowest percent free saccharide recovery for the conjugated samples tested at <2% and slightly higher results for DOC (4% for MenA-CRM_197_ samples and 3–11% for MenW-CRM_197_ samples) (Table 4 and Table 5).

## 4. Discussion

Glycoconjugate vaccines protect against diseases caused by encapsulated bacteria by generating bactericidal antibodies specific to the bacterial PS. Ensuring that the polysaccharide remains conjugated to the protein and that unconjugated or “free” saccharide levels are minimal is crucial for maintaining vaccine potency and immunogenicity [43,44]. Many methods have been explored for conjugate purification and analysis, including “free” saccharide separation and quantitation [7].

The methods employed for the separation and subsequent free saccharide quantitation depend on the nature of the protein conjugate and the PS [7], which in turn are reliant on their solution structure and behavior [45,46,47,48]. Indeed, different studies have emphasized the importance of the protein [45], while others emphasized the polysaccharide component [41,46] in dominating the solution behavior of the glycoconjugate. The PS chain (both native and chemically activated) can present a surface electrostatic charge and can be characterized by a range of molecular sizes (hydrodynamic volume and chain length), molecular masses (M_w_), and molecular dynamics. These factors, and the presence of substituent groups such as *O*-acetyl, may impact the shape, chemical nature, flexibility, and viscosity of PS solutions and can make physical separations more difficult, leading to challenges. In the case of the group C meningococcal PS, its relatively high flexibility [46,47,49] may facilitate more efficient transmission across ultrafiltration membranes relative to other serogroups.

The ultrafiltration method relies on the size-based separation of free *saccharide*, yet the membranes used for this type of separation are typically characterized by their ability to separate *proteins* of nominal sizes, for example, 10, 30, and 100 kDa. Their “cutoff” size values are used for selecting the most appropriate membranes to retain the carrier proteins (and conjugated saccharide) but are not informative in understanding the membrane retention or filterability of poly- or oligosaccharides. While it is essential that the selected membrane retains any conjugated carrier protein, for which the cutoff values are useful (for example, the choice of a 30 kDa membrane to retain the carrier protein CRM_197_ (58.4 g/mol), and the choice of a 100 kDa membrane to retain tetanus toxoid (155–163 g/mol) [36,50], a relatively large pore size for allowing for the filtration of unconjugated saccharide is generally desired.

The results obtained with the 30 kDa membrane suggest that the upper size limit of MenC PS that can pass through in a buffered solution is approximately 20,000 g/mol, whereas the upper PS size limit for the 100 kDa membrane was found to be approximately 53,000 g/mol (Table 1 and Figure 2a). These findings indicate that the method is ideally suited for determining the stability of glycoconjugate vaccines and performing lot control testing. Thus, the depolymerization and hydrolysis of end-of-chain groups releasing small oligosaccharides can be monitored, rather than measuring the content of large polysaccharides that remain unconjugated after manufacture, such as may occur in large TT conjugates that form cross-linked complexes. The relatively shorter chains typical of sun-type monomeric CRM_197_ conjugates would be expected to pass through the filters. The UF method used in this study has been validated for evaluating the free saccharide in CRM_197_ and TT conjugates when depolymerization leads to free saccharide impurities [17,31,32]. Our results may also aid in the selection of membranes for filtering polysaccharides in a manufacturing setting when considering the adequacy of pore size, with further consideration of the ionic strength, PS charge, and flexibility.

Previous studies have investigated the effects of physical factors on the size and movement of charged bacterial polysaccharides through chromatography columns and ultrafiltration membranes and identified ionic strength as an important determinant for the purification and concentration of polysaccharides used for glycoconjugate vaccine production [39,40,41,51]. Specifically, PS transmission through an ultrafiltration membrane in dilute solutions decreased with decreasing ionic strength arising from the increase in the effective size of charged polysaccharides due to charge repulsion. Conversely, raising the ionic strength increased the transmission of the charged polysaccharides because of their smaller size arising from the shielding of intermolecular electrostatic interactions [39,41].

This can be used in two ways: a low solution ionic strength could be used to increase the retention of charged PS in a UF step used for PS concentration (where high retention is desired), while increased ionic strength could be used to enhance the removal of free (unconjugated/unreacted) PS in the preparation of conjugated vaccines. The starting concentration of PS in the UF module and the flow applied through the UF membrane could have an impact that is tightly interconnected with solution ionic strength and pH [39,40]. In addition, the nature of the membrane in terms of electrostatic and hydrophobic interactions with charged polysaccharides and proteins, as well as the surface charge of the membrane material itself, could have an impact on transmission through the membrane [39,40,52], the geometry of the UF module, and the flow rate applied through the membrane [52]. Membrane fouling due to PS or PS–protein conjugate adsorption on the membrane or the formation of a gel layer can reduce UF efficiency due to a physical blockage of the pores. The adjustment of operating conditions, such as transmembrane pressure and crossflow velocity, can enhance UF performance by mitigating fouling and optimizing polarization effects and shear rates [53]. All these parameters that alter the PS conformation and interactions (both intramolecular and intermolecular, but also with the protein and the UF membrane) need to be considered in selecting the membrane and pore size of a UF membrane, in relation to the purpose of the UF step itself (retention vs. permeation), with the final aim of optimizing filtration processes in biotechnology.

The effect of solution conditions (pH and ionic strength) was also observed with size-exclusion chromatography of charged bacterial polysaccharides and their glycoconjugates [41]. Men A, C, W, X, and Y polysaccharides were previously shown to swell in water, relative to buffered solutions, with increases of greater than 10 nm hydrodynamic radii observed in water [54]. In this study, no difference was observed in the amount or recovery of free saccharide in a buffered solution due to increasing ionic strength up to 250 mM KCl or, conversely, when water was a diluent, probably due to residual salts present from the concentrated bulk conjugate. Deeper investigations are needed to better understand the role of the ionic strength on the behavior of bacterial polysaccharide solutions in both UF and SEC methodologies as applied to real-world manufacturing and analytical laboratory conditions. This study did not explore the impact of *O*-acetylation on the shape and flexibility of the polysaccharide, but the impacts of *O*-acetylation on conformation, viscosity, and filtration have been observed in groups A and C meningococcal and other saccharide types such as typhoid Vi [54,55,56,57].

The ultrafiltration method has been validated for use in the control testing of various different saccharide types used in glycoconjugate vaccines, including Hib, meningococcal (A, C, W, X, Y), and typhoid polysaccharides, and results with a stability evaluation of the conjugates have shown its usefulness [17,31,32]. This study focused on meningococcal group C, with additional analysis using groups A and W. Each of these has different charge densities, saccharide chain loading, flexibilities, and behavior [46,47], and this may be why relatively different levels of % free saccharide were measured by the various free saccharide methods compared in this study. MenC is one of the more flexible meningococcal serogroups [46], and it has been variously described as a flexible, random coil with a propensity to self-associate in high concentrations [49], or a zig-zag ribbon that “breathes” [47]. When the UF method was used, a higher percent of free MenC was found in the CRM_197_-conjugated vaccine studied here and in a separate MenC-CRM_197_ conjugate in a pentavalent meningococcal vaccine [32] relative to other serogroups, yet the DOC precipitation method measured lower free MenC than the other serogroups. MenC glycoconjugates showed very different free saccharide percentages using the three methods used in this study (7–8% for UF, less than 1% for DOC, and up to 15% for SPE, which yielded the highest variability).

Each of the free saccharide methods has advantages, drawbacks, and specific biases. Challenges with the free saccharide assay faced by one company are discussed in a review in this journal issue [8]. The UF method has limitations as a size-based method but is non-denaturing, and both free and total saccharide can be measured in the same assay.

The high recovery of the low MW polysaccharide control as demonstrated is an advantage of the detergent precipitation method, but the direct addition of acid and the need to keep samples cool during processing can be a drawback. Detergent precipitation is a relatively fast method but requires careful consideration of the carrier protein and the optimization of the pH and detergent concentration; additionally, it is important to assess whether the detergent interferes with saccharide quantification [12,13,58].

The C4 bead binding (or SPE) method, which relies on the hydrophobicity of the protein carrier, is one of the most complex of the methods and requires optimization for each of the serogroups and the carrier protein, with respect to the SPE column resin and conditioning procedure, to allow the free saccharide component to pass through the beads without binding whilst capturing the conjugated saccharide [21]. The hydrophobic beads or matrices of the SPE cartridges were a likely cause of the poorer recovery of the low MW MenC control in this study. The efficiency of the binding of the conjugated protein may, in theory, also lead to an overestimation of free saccharide relative to other methods.

Polysaccharides are generally considered to be hydrophilic molecules due to their solubility in aqueous solvents with favorable electrostatic interactions, and solvent complexation. The hydroxyl groups and ring oxygen atom on a saccharide unit of a PS can form hydrogen bonds with neighboring saccharide residues, resulting in intramolecular interactions; water also can serve as a hydrogen bond donor and acceptor and can compete with the intramolecular interactions and shield the PS from long-range electrostatic interactions [45]. However, under certain conditions, factors relating to higher PS concentrations, particular glycosidic linkages, and substituent groups; complexation of the negatively charged meningococcal polysaccharides with calcium or other divalent cations; the water hydration sphere and the presence of specific solutes and excipients; and the proximity of hydrophobic protein regions or nonpolar matrices may lead to the formation of local areas with polysaccharide hydrophobicity, which can lead to undesired solution behavior, insolubility, and irreversible binding to surfaces as observed with typhoid Vi polysaccharide [8]. The hydrophobic nature of PS, under these conditions, can also increase with chain length. The longer chain provides more surface area for potential hydrophobic interactions [59,60]. The size and the length of the PS can have an impact on the binding and elution from the cartridge; enhanced binding of unconjugated longer chain polysaccharide to the SPE matrix together with the conjugate may occur, whereas smaller fragments may be more easily eluted.

With all of the methods, the conditions promoting the self-association of group C polysaccharide or association with the conjugate or other components should be avoided, and adequate ionic strength should be considered to improve solution behavior.

In 1997, during a WHO consultation on the requirements for the manufacturing and control of Hib vaccines, it was agreed that without a valid animal model for Hib conjugates, the most important measure of dose was the amount of “conjugated saccharide”. The recommendations for MenC conjugates state that “only the polysaccharide that is covalently bound to the carrier protein (i.e., conjugated polysaccharide) is immunologically important for clinical protection and excessive levels of unbound polysaccharide could potentially result in immunological hypo-responsiveness” [44]. Each batch of conjugate is therefore assessed for free and total saccharide. In evaluating glycoconjugate vaccines, the common approach is to determine both the free saccharide content and the total saccharide as separate fractions in order to determine the percentage of free saccharide. This has been further rationalized to evaluate the conjugated content, i.e., the effective dose. One approach has been to first remove the free saccharide element using a 30 kDa ultrafiltration membrane and subsequently recover and quantify the conjugated material from the retentate of the filter [18]. Consistency between the free saccharide analysis by SPE and ultrafiltration was confirmed for meningococcal group A [18]. Additional studies with such approaches to measure the “conjugated saccharide” are encouraged for products under development so that the glycoconjugate community can adopt a simpler and more direct measure of the dosage. Consideration of the use of other membrane materials, which may provide alternative pore sizes, such as with 50 kDa or larger pore size membranes and more selective pore sizes, could be helpful [61]. This study was a preliminary study, but it highlights the importance of understanding the polysaccharide size, shape, and solution behavior in improving methodologies for production and analysis. The eventual call to measure the “conjugated saccharide” will test the usefulness of the current methodology for new products.

Through further exploration of the size-dependent ultrafiltration method to determine the amount of “free” or unconjugated meningococcal saccharide present in glycoconjugate vaccines, the present study extends our knowledge of the significance of the hydrodynamic size of PS in relation to membrane pore size and represents, to our knowledge, the first study on the size characterization of meningococcal PS applied to filtration using regenerated cellulose (low binding) membranes. It serves to enhance our understanding of the role of this analytical approach and situates it with other methods to ensure the integrity and, ultimately, the potency of glycoconjugate vaccines.

## Figures and Tables

**Table 1:** Size characteristics of meningococcal C polysaccharide fragments.

Sample	Mw (g/mol)	Polydispersity (Mw/Mn)	Hydrodynamic RMS Radius Moments (Rn, nm)
*MenC polysaccharides in ultrafiltration membrane characterization*			
MenC (FL) 0 min	287,300 (±8%)	1.03 (±11%)	19.5 (±4%)
MenC 15 min	130,000 (±5%)	1.09 (±8%)	13.3 (±3%)
MenC 30 min	52,860 (±4%)	1.07 (±20%)	7.3 (±8%)
MenC 90 min	22,190 (±28%)	1.51 (±52%)	3.7 (±22%)
Low MW control used in free saccharide assays *			
MenC control	24,100	1.11	8.4

* Lockyer et al., 2020 [37]. The values in parentheses represent the instrument error as reported by the Astra version 6.1 software.

**Table 2:** Percentage of free saccharide following ultrafiltration of MenC-CRM_197_ conjugates at varying ionic strengths.

Diluent	Free Saccharide (%)
Water	7.5 (%CV 8.7)
10 mM Bis-Tris, pH 7	6.5 (%CV 3.5)
5 mM KCl in 10 mM Bis-Tris, pH 7	6.8 (%CV 4.1)
50 mM KCl in 10 mM Bis-Tris, pH 7	6.3 (%CV 8.6)
250 mM KCl in 10 mM Bis-Tris, pH 7	6.2 (%CV 0.7)

The %CV values of the free saccharide were determined from three replicate preparations.

**Table 3:** Comparison of free saccharide methods, ultrafiltration (UF), detergent precipitation (DOC), and hydrophobic beads (SPE) for MenC-CRM_197_ conjugates.

Method	Sample	Replicate	Free Saccharide (%)—DAY 1		Free Saccharide (%)—DAY 2		Free Saccharide (%)—Combined	%CV
			% Free	Average	% Free	Average	Average	
UF	C	1	5.5	6.8	5.3	6.1	6.5	0.65
		2	8.6		6.7			
		3	6.4		6.3			
	D	1	6.7	6.7	9.2	10.0	8.4	1.4
		2	8.8		10.1			
		3	4.6		10.7			
	Low MW MenC control	1	19.4	-	20.0	-	19.7	-
DOC	C	1	1.2	1.1	0.3	0.4	0.8	0.4
		2	1.1		0.5			
		3	1.0		0.4			
	D	1	1.1	1.1	0.3	0.3	0.7	0.4
		2	1.0		0.3			
		3	1.2		0.3			
	Low MW MenC control	1	90.8	-	98.3	-	94.6	-
SPE	C	1	11.8	15.0	0.3	14.5	14.8	9.0
		2	15.1		18.1			
		3	18.1		25.1			
	D	1	11.1	11.6	9.0	18.6	15.1	3.6
		2	13.1		20.7			
		3	10.5		26.0			
	Low MW MenC control	1	75.5	-	-	-	75.5	-

**Table 4:** Comparison of free saccharide methods, ultrafiltration (UF), and detergent precipitation (DOC) for MenA-CRM_197_ conjugates.

Method	Sample	Replicate	Free Saccharide (%)—DAY 1		Free Saccharide (%)—DAY 2		Free Saccharide (%)—Combined	%CV
			% Free	Average	% Free	Average	Average	
UF	A	1	1.9	1.8	2.1	2.0	1.9	0.1
		2	1.9		2.0			
		3	1.7		2.0			
	B	1	1.3	1.2	1.2	1.2	1.2	0.0
		2	1.2		1.3			
		3	1.2		1.2			
	Low MW MenA control	1	33.1	-	40.3	-	36.7	-
DOC	A	1	4.7	4.5	4.2	3.8	4.1	0.3
		2	4.7		4.0			
		3	4.0		3.3			
	B	1	4.0	4.0	4.2	4.3	4.2	0.1
		2	4.0		4.6			
		3	4.0		4.1			
	Low MW MenA control	1	102.5	-	106.6	-	104.5	-

**Table 5:** Comparison of free saccharide methods, ultrafiltration (UF), and detergent precipitation (DOC) for MenW-CRM_197_ conjugates.

Method	Sample	Replicate	Free Saccharide (%)—DAY 1		Free Saccharide (%)—DAY 2		Free Saccharide (%)—Combined	% CV
			% Free	Average	% Free	Average	Average	
UF	E	1	0.5	0.7	0.6	0.7	0.7	0.1
		2	1.0		0.7			
		3	0.6		0.7			
	F	1	0.7	1.0	0.9	0.8	0.9	0.1
		2	0.9		0.6			
		3	1.5		1.1			
	Low MW MenW control	1	34.9	-	40.6	-	37.7	-
DOC	E	1	3.3	3.2	2.2	2.0	2.6	0.6
		2	3.5		1.8			
		3	2.9		1.9			
	F	1	7.8	8.1	13.4	13.8	11.0	3.2
		2	8.2		15.1			
		3	8.3		13.0			
	Low MW MenW control	1	103.3	-	90.4	-	96.9	-

## Data Availability

The raw data supporting the conclusions of this article will be made available by the corresponding authors on request.
